# Regulation of Oxidative Stress by Long Non-coding RNAs in Central Nervous System Disorders

**DOI:** 10.3389/fnmol.2022.931704

**Published:** 2022-06-15

**Authors:** Xiaoman Xu, Yi Zhang

**Affiliations:** ^1^Department of Pulmonary and Critical Care Medicine, Shengjing Hospital of China Medical University, Shenyang, China; ^2^Department of Gerontology and Geriatrics, Shengjing Hospital of China Medical University, Shenyang, China

**Keywords:** long non-coding RNAs, oxidative stress, therapeutic target, central nervous system, pathogenesis

## Abstract

Central nervous system (CNS) disorders, such as ischemic stroke, Alzheimer’s disease, Parkinson’s disease, spinal cord injury, glioma, and epilepsy, involve oxidative stress and neuronal apoptosis, often leading to long-term disability or death. Emerging studies suggest that oxidative stress may induce epigenetic modifications that contribute to CNS disorders. Non-coding RNAs are epigenetic regulators involved in CNS disorders and have attracted extensive attention. Long non-coding RNAs (lncRNAs) are non-coding RNAs more than 200 nucleotides long and have no protein-coding function. However, these molecules exert regulatory functions at the transcriptional, post-transcriptional, and epigenetic levels. However, the major role of lncRNAs in the pathophysiology of CNS disorders, especially related to oxidative stress, remains unclear. Here, we review the molecular functions of lncRNAs in oxidative stress and highlight lncRNAs that exert positive or negative roles in oxidation/antioxidant systems. This review provides novel insights into the therapeutic potential of lncRNAs that mediate oxidative stress in CNS disorders.

## Introduction

Central nervous system (CNS) disorders, such as acute ischemic stroke (AIS), Alzheimer’s disease (AD), Parkinson’s disease (PD), spinal cord injury (SCI), glioma, and epilepsy, usually lead to serious clinical consequences, long-term disability, or death (Xu et al., 2021). Several pathological processes involved in CNS disorders, including neuroinflammation, mitochondrial dysfunction, apoptosis, oxidative stress, and autophagy result in impaired CNS structure and dysfunction (Anderson et al., 2016; Khoshnam et al., 2017). In these pathological processes, oxidative stress plays a pivotal role in each disease (Scannevin et al., 2012). Oxidative stress refers to a pathological state in which free radicals in the body exceed its antioxidant capacity. Due to the redox imbalance, excessive reactive oxygen species (ROS) and reactive nitrogen (RNS) are generated, leading to Fenton reactions occurring *via* the action of metal ions to form hydroxyl radicals (⋅OH) (Sies, 2015; Sinha and Dabla, 2015). Excessive ROS induces lipid peroxidation and DNA, RNA, and protein oxidation, leading to neuronal dysfunction and death (Chen et al., 2011; Ouyang et al., 2015). Previous studies suggest that patients with higher concentrations of lipid peroxidation mediators have worse prognosis in CNS disorders, such as in AIS and AD (Klimiuk et al., 2019; Maciejczyk et al., 2020). Therefore, considering that hyperactive oxidative stress responses arise after the occurrence of CNS disorders, finding effective strategies to modulate oxidative stress in the CNS is important for restricting oxidative injuries and protecting neurological function (Costa et al., 2016).

Emerging studies suggest that oxidative stress may induce epigenetic modifications that ultimately lead to CNS disorders (Zhao et al., 2016). Non-coding RNAs have attracted extensive attention as epigenetic regulators involved in CNS disorders (Wu and Kuo, 2020). Long non-coding RNAs (LncRNAs) are a class of RNAs more than 200 nucleotides long but have no protein-coding function. These molecules were initially considered as a transcription by-product without important biological functions. However, numerous studies found that lncRNAs serve as an important “medium” in cells (Hombach and Kretz, 2016). LncRNAs are involved in the regulation of cell proliferation, differentiation, the cell cycle, and apoptosis at the transcriptional, post-transcriptional, and epigenetic levels. Furthermore, a few annotated lncRNAs play an important role in oxidative stress-related diseases and CNS disorders (Chu et al., 2022). For instance, elevated levels of the lncRNA rhabdomyosarcoma 2 related transcript (RMST) augment AIS by reducing microRNA (miR)-221-3p-mediated regulation of phosphoinositide-3-kinase regulatory subunit 1 (PIK3R1) and activating the transforming growth factor-β (TGF-β) pathway (Li et al., 2022). In contrast, knocking down lncRNA Gm11974 attenuates neuronal injury in AIS by modulating the miR-122-5p/semaphorin 3A (SEMA3A) signaling pathway (Yang et al., 2021). These results reveal that lncRNAs exert a positive or negative role in oxidative stress responses and suggest that lncRNAs may be key molecules involved in oxidative stress.

Despite recent advances, the role of lncRNAs and their downstream regulatory networks in regulating oxidative stress remains unclear. In this review, we collect existing evidence and discuss the characteristics of lncRNAs and their involvement in the oxidative/antioxidant system in different CNS disorders including AIS, neurodegenerative diseases, traumatic diseases, epilepsy, and glioma. Moreover, we discuss the potential molecular mechanisms involved in the regulation of oxidative stress, which may provide new insights into potential therapeutic lncRNA targets in CNS disorders that mediate oxidative stress.

## Oxidative Stress and the Nrf2/Keap1/Are Pathway

**FIGURE 1:**
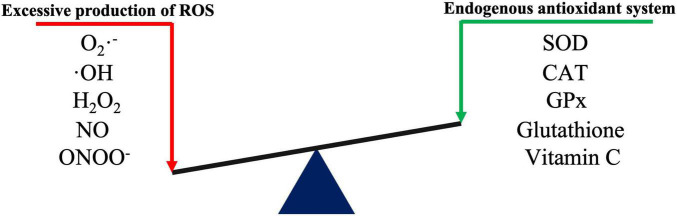
Schematic diagram of redox reactions.

Oxidative stress occurs when the physiological balance between oxidants and antioxidants is disrupted, which shifts the balance to favor oxidants and results in potential damage to the body (Sies et al., 2017). Oxidative stress and the related inflammatory responses, autophagy and apoptosis, are key factors involved in CNS disorders (Hybertson et al., 2011). ROS are continuously produced by all aerobic organisms through both enzymatic and non-enzymatic reactions. The most common ROS include superoxide anion radicals (O_2_⋅^–^), hydroxyl radicals (⋅OH), hydrogen peroxide (H_2_O_2_), nitric oxide (NO), and nitrite peroxide (ONOO^–^). In humans, the major ROS sources are mitochondria and various ROS-producing enzymes, including nicotinamide adenine dinucleotide phosphate (NADPH) oxidase (NOX), xanthine oxidase (XO), nitric oxide synthase (NOS), and myeloperoxidase (MPO) (Pizzino et al., 2017; Jakubczyk et al., 2020). The CNS has a relatively poor antioxidant defense due to its high oxygen consumption and high polyunsaturated fatty acid levels. At the physiological level, neurons are repaired through their own antioxidant defense system, which prevents neuronal oxidative damage (Salim, 2017). When excessive ROS production exceeds the repair capacity of the endogenous antioxidant system, biological macromolecules (such as lipids, proteins, and nucleic acids) undergo oxidative damage and can even activate apoptosis. Endogenous antioxidant enzymes such as superoxide dismutase (SOD), catalase (CAT) and glutathione peroxidase (GPx), and non-enzymatic antioxidants such as glutathione, ubiquinone, and ascorbic acid (vitamin C) help maintain cellular redox homeostasis (Figure 1; Rodrigo et al., 2013; Salim, 2017).

Among the antioxidant defense mechanisms, nuclear factor-erythroid related factor-2 (Nrf2) is a master regulator of transcriptional activation in antioxidant effects and can balance ROS production. Under oxidative stress, Nrf2 dissociates from Kelch-like Ech-associated protein 1 (Keap1) and translocates into the nucleus, where it binds to antioxidant response elements (ARE) (Silva-Palacios et al., 2018), thereby activating downstream antioxidant defense enzymes such as NAD(P)H quinone dehydrogenase 1 (NQO1), heme oxygenase 1 (HO-1), glutathione S-transferase (GST), and enzymes involved in glutathione synthesis and metabolism (γ-glutamyl cysteine synthetase) (Yamamoto et al., 2018; Baird and Yamamoto, 2020). Unfortunately, decreased antioxidant proteins have been found in many CNS disorders. Recent findings on the association of lncRNAs with oxidative stress may provide new ideas for explaining this phenomenon.

## LncRNA Classification

**FIGURE 2:**
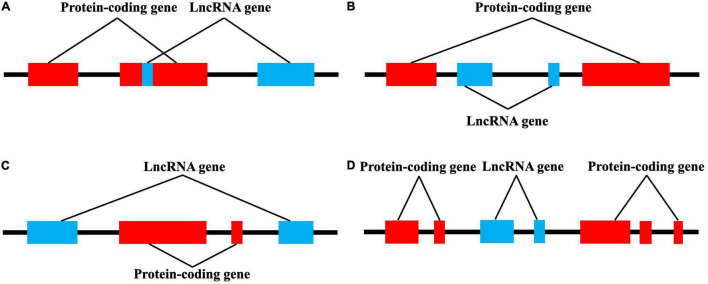
lncRNAs are classified into four categories by their location relative to neighboring protein-coding genes. **(A)** Exon lncRNAs, **(B)** intron lncRNAs, **(C)** overlapping lncRNAs, and **(D)** intergenic lncRNAs.

LncRNAs account for 80–90% of all ncRNAs. Compared with other ncRNAs such as microRNAs (miRs) and circular RNAs, lncRNAs have longer sequences, more complex spatial structures, and more diverse and complex mechanisms involved in the regulation of gene expression (Esteller, 2011). LncRNAs generally have similar characteristics to protein-coding genes, but tend to contain only one intron and have a low tendency for co-transcriptional splicing. Similar to mRNA, lncRNAs are alternately spliced and are primarily transcribed by RNA polymerase II, with about half having 5′-Cap and 3′-polyadenosine structures. LncRNAs also have a special secondary structure that provides several protein and DNA/RNA binding sites (Wei et al., 2018). Thus, lncRNAs regulate gene expression in various ways: (i) by interfering with transcription factor binding to target genes; (ii) interacting with small RNA; (iii) binding to proteins and acting as ribonuclein scaffolds; (iv) binding to chromatin to regulate chromatin remodeling; (v) and binding mRNA and affecting translation, shearing, and degradation (Shi et al., 2013; Deniz and Erman, 2017). LncRNAs have differences in size, molecular partners, and mechanism of action. According to their relative position and host protein-coding genes, lncRNAs can be divided into exons, introns, overlapping lncRNAs, and intergenic lncRNAs (Figure 2; Tan et al., 2021).

**FIGURE 3:**
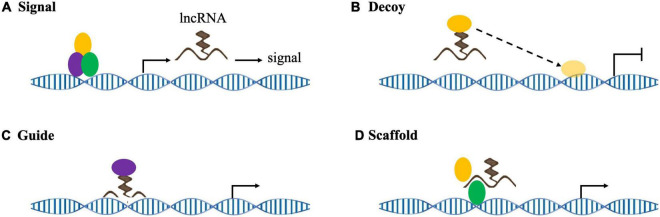
lncRNA mechanisms of action. **(A)** As signaling molecules, lncRNAs convey specific cell development and spatiotemporal information to regulate corresponding gene expression. **(B)** As decoy molecules, lncRNAs sequester target molecules, which inhibits downstream functions. **(C)** As guide molecules, lncRNAs recruit chromatin modification complexes to target genes in either *cis* or *trans*. **(D)** As scaffold molecules, lncRNAs form functional complexes to participate in histone modification and/or stabilize nuclear structures.

There is no clear, unified standard for lncRNA classification. Indeed, lncRNA can be basically divided into the following categories based on their relative positions with encoding genes: intergenic lncRNA (lincRNA), sense lncRNA, antisense lncRNA, untranslated lncRNA, promoter-related lncRNA (pancRNA), introns lncRNA (intronic RNA), and enhancing lncRNA. The position of the lncRNA in the genome often determines its regulating mechanism and related functions (Chen et al., 2021a). In addition, lncRNAs are roughly classified into four categories according to their roles: signal molecules, decoy molecules, guide molecules, and scaffold molecules (Figure 3; Wang and Chang, 2011). As signal molecules, lncRNAs participate in the conduction of some signal pathways. Some lncRNAs regulate the transcription of downstream genes and reflect their spatiotemporal expression. As decoy molecules, lncRNAs can combine with and remove some transcription factors to regulate gene expression. As guide molecules, lncRNAs can recruit *cis*- or *trans*- genes for chromatin modification enzymes. As scaffold molecules, lncRNAs can bind various proteins to form complexes and modify histones on chromatin (Dahariya et al., 2019; He et al., 2021).

The functions of lncRNAs are well known and numerous abnormal lncRNAs are observed in CNS disorders. However, research on the mechanism and possible consequences of abnormal lncRNAs in CNS disorders remains limited, especially for CNS disorders involving oxidative stress. In this review, we specifically focus on how lncRNAs regulate oxidative stress in CNS disorders (Table 1). By mediating oxidative stress, lncRNAs may provide new potential targets for the treatment of CNS disorders.

**TABLE 1:** The molecular targets, downstream pathways and oxidative stress regulation of lncRNAs in central nervous system (CNS) disorders.

CNS disorders	lncRNAs	Expression	Intermediate molecule	Downstream pathway	Animals or Cells	Models	Observed oxidative stress indicators	References
AIS	NEAT1	Decreased	Mfn2	Sirt3	BV-2/N2a cells	OGD/R	ROS, SOD, MDA	Zhou et al., 2022
	RMST	Increased	miR-377	SEMA3A	N2a cells	OGD/R	ROS, MDA, SOD, NO	Zhao et al., 2021
	AK139328	Increased	Netrin-1	NA	PC12 cells	OGD/R	ROS, eNOS	Liu et al., 2021
	SNHG14	Increased	miR-199b	AQP4	BV-2 cells	OGD/R	SOD, MDA	Liu et al., 2021
	MACC1-AS1	Decreased	miR-6867-5p	TWIST1	HBMECs	Hypoxia	ROS, SOD, MDA, CAT	Yan et al., 2020
	OIP5-AS1	Decreased	miR-186-5p	CTRP3	Rats/BV-2 cells	MCAO/R	MDA, SOD, GSH-Px	Chen et al., 2021b
	CEBPA-AS1	Increased	miR-24-3p	BOK	SH-SY5Y cells	OGD/R	ROS, SOD, GSH	Di et al., 2021
	KCNQ1OT1	Increased	miR-140-3p	HIF-1α	PC12 cells	OGD/R	ROS, SOD, MDA, LDH	Yi et al., 2020
	ZFAS1	Decreased	miR-582-3p	NOS3	PC12 cells	OGD/R	MDA, LDH, GSH-px, SOD, NO, eNOS	Zhang and Zhang, 2020
	SNHG16	Decreased	miR-421	XIAP	SK-N-SH cells	OGD/R	ROS, SOD, MDA, LDH	Cao et al., 2022
	GAS5	Increased	miR-455-5p	PTEN	Rats/PC12 cells	OGD/R, MCAO/R	CAT, SOD, GSH-Px	Wu et al., 2021
	Gm11974	Increased	miR-122-5p	SEMA3A	Mice/N2a cells	OGD/MCAO	MDA, LDH, NO, CAT, H2O2	Yang et al., 2021
	SNHG7	Decreased	miR-134-5p	FGF9	N2a cells	OGD	ROS, SOD, MDA, CAT, LDH	Sun et al., 2021
	AK046177	Increased	miR-134	CREB	Rats/Primary cortical cells	OGD/R/MCAO	SOD, GSH-Px, MDA, NADPH, Nrf2	Wang et al., 2020a
AD	XIST	Increased	miR-132	NA	Hippocampal neurons	Aβ_25–35_	SOD, GSH-Px, MDA	Wang et al., 2018b
	H19	Increased	miR-129	HMGB1	PC12 cells	Aβ_25–35_	SOD, MDA, CAT	Zhang et al., 2021
	BDNF-AS	Increased	NA	BDNF	PC12 cells	Aβ_25–35_	SOD, MDA, CAT, ROS	Guo et al., 2018
	WT1-AS	Decreased	WT1	miR-375/SIX4	SH-SY5Y cells	Aβ_25–35_	ROS, MDA, LDH, SOD, GSH-Px	Wang et al., 2020b
	TUG1	Increased	miR-15a	ROCK1	Mice/Hippocampal neurons	Aβ_25–35_	MDA, SOD	Li et al., 2020
PD	MIAT	Increased	miR-221-3p	TGF-β1/Nrf2 axis	Mice/MN9D dopaminergic neuronal cells	MPTP	SOD, GSH, MDA	Lang et al., 2022
	NORAD	Decreased	miR-204-5p	SLC5A3	Neuroblastoma/SK-N-SH/-N-AS cells	MPP^+^	SOD, LDH	Zhou et al., 2020
	RMST	Increased	NA	TLR/NF-κB signaling	Rats	MPTP	SOD, CAT, GSH-Px, NOS, MDA, NO	Ma et al., 2021
	AL049437	Increased	miR-205-5p	MAPK1	Mouse/SH-SY5Y cells	MPTP/MPP^+^	ROS	Zhang et al., 2020a
	MALAT1	Increased	EZH2	Nrf2	C57BL/6 mice	MPTP	SOD, CAT	Cai et al., 2020
	Lnc-p21	Increased	miR-625	TRPM2	SH-SY5Y	MPP^+^	SOD	Ding et al., 2019
	T199678	Decreased	miR-101-3p	α-Syn	SH-SY5Y cells	α-Syn	ROS	Bu et al., 2020
SCI	CASC9	Decreased	miR-383-5p	LDHA	Rats/PC12 cells	LPS/Pentobarbital	LDH, MDA	Guan and Wang, 2021
	GAS5	Increased	CELF2	VAV1	RN-Sc cells	OGD/R	GSH-Px, SOD, MDA	Wang et al., 2021a
	TCTN2	Decreased	miR-329-3p	IGF1R	Rats/PC12 cells	LPS	SOD, MDA	Liu et al., 2022
	SOX2OT	Increased	miR-331-3p	Neurod1	Rats/PC12 cells	LPS	SOD, MDA	Li et al., 2021b
Glioma	H19	Increased	NA	NA	U251/LN229 cells	H_2_O_2_	NA	Duan et al., 2018
TLE	MEG3	Decreased	NA	PI3K/AKT/mTOR pathway	Rats	LiCl/Pilocarpine	SOD, MDA	Zhang et al., 2020a

*α-Syn, α-synuclein; AD, Alzheimer’s disease; AIS, acute ischemic stroke; CAT, catalase; eNOS, endothelial nitric oxide synthase; EZH2, enhancer of zeste homolog 2; GSH-PX, glutathione peroxidase; HBMECs, hypoxia-induced human brain microvascular endothelial cells; LDH, lactate dehydrogenase; LPS, lipopolysaccharide; MCAO/R, middle cerebral artery occlusion/reperfusion; MDA, malondialdehyde; MPP^+^, 1-Methyl-4-phenylpyridinium ion; MPTP, 1-methyl-4-phenyl-1,2,3,6-tetrahydropyridine; NADPH, nicotinamide adenine dinucleotide phosphate; NF-κB, nuclear factor kappa B; NO, nitric oxide; NOS3, nitric oxide synthase 3; Nrf2, nuclear factor E2-related factor 2; OGD/R, oxygen-glucose deprivation/reoxygenation; PD, Parkinson’s disease; RN-Sc, rat neurons-spinal cord; ROS, reactive oxygen species; SCI, spinal cord injury; SOD, superoxide dismutase; TGF-β1, transforming growth factor-β1; TLE, temporal lobe epilepsy; TLR, Toll-like receptor.*

## LncRNAs Mediating Oxidative Stress in Central Nervous System Disorders

### LncRNAs Mediating Oxidative Stress in Acute Ischemic Stroke

**FIGURE 4:**
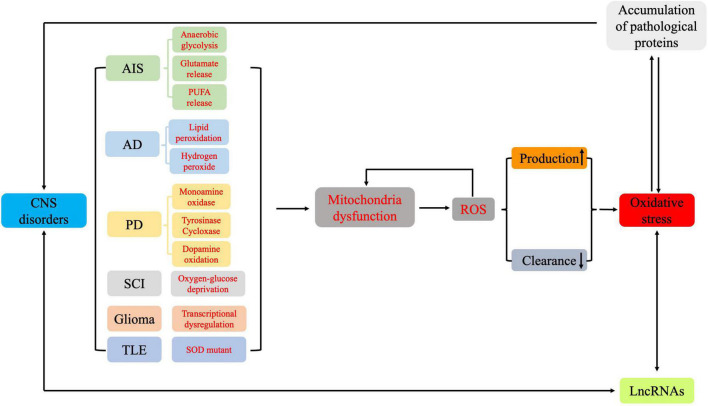
The pathways involved in oxidative stress and in the formation of reactive oxygen species in the different pathologies of central nervous system disorders.

During AIS, the sudden reduction or interruption of glucose and oxygen supply rapidly disturbs energy metabolism in brain tissue, which leads to the generation of abundant ROS and oxidative intermediates, resulting in severe oxidative damage in a short period of time (Wang et al., 2018a). In early AIS, cellular metabolism shifts to anaerobic glycolysis, resulting in decreased NADPH, increased O_2_^–^, and dysregulated neuronal energy metabolism (Burmistrova et al., 2019). This also causes neuronal ion channels to malfunction and cell membranes to depolarize, leading to excessive release of excitatory transmitters and subsequent excitatory toxicity (Amantea and Bagetta, 2017). Excessive glutamate release causes Na^+^–Ca^2+^ exchanger (NCX) dysfunction and mitochondrial depolarization, resulting in calcium overload and increased ROS production (Shi et al., 2018). Additionally, phospholipase is activated, which leads to polyunsaturated fatty acid (PUFA) release and ROS generation as PUFAs are metabolized into inflammatory mediators such as prostaglandins (Chuang et al., 2015; Figure 4).

Nuclear paraspeckle assembly transcript 1 (NEAT1) is a lncRNA that is dysregulated in various human cancers (Li et al., 2021a). NEAT1 plays a protective role in oxygen-glucose deprivation/reoxygenation (OGD/R)-activated brain microvascular endothelial cells (Zhou et al., 2019). As an activator of the antioxidant pathway, NEAT1 overexpression stabilizes Mfn2 mRNA by recruiting Nova, thereby increasing Mfn2 expression and alleviating ischemia-reperfusion-induced oxidative stress and apoptosis *via* the Mfn2/Sirt3 pathway (Zhou et al., 2022).

The lncRNA rhabdomyosarcoma 2-associated transcript (RMST) has pivotal roles in regulating AIS through multiple pathophysiological mechanisms, including oxidative stress. In OGD/R-induced AIS, increased RMST was observed. Downregulating RMST ameliorated increased MDA and ROS while decreasing SOD and NO levels. The effect of RMST downregulation abrogates OGD/R-triggered oxidative stress, likely by downregulating SEMA3A expression *via* sponging miR-377 (Zhao et al., 2021).

Previous studies reported that the lncRNA AK139328 is associated with ischemia/reperfusion injury (IRI) in various organs (Chen et al., 2015; Yu et al., 2018). AK139328 is increased in PC12 cells activated by OGD/R. However, AK139328 silencing decreases ROS production and upregulates endothelial nitric oxide synthase (eNOS) protein expression, which suggests that knocking down AK139328 may alleviate OGD/R-induced oxidative stress in PC12 cells (Liu et al., 2021).

OGD could upregulate lncRNA small nucleolar RNA host gene 14 (SNHG14) and downregulate its derived miR-199b in BV2 cells. SNHG14-derived miR-199b targets the 3′ UTR of aquaporin 4 (AQP4) to increase SOD activity and markedly decrease MDA levels (Liu et al., 2021).

MACC1-AS1 is the antisense lncRNA of MACC1 and has been identified as an oncogene in multiple cancers (Zhao et al., 2018; Guo et al., 2020). miR-6867-5p suppresses the proliferation of endometriosis (Park et al., 2019); MACC1-AS1, the endogenous competitor of miR-6867-5p, exerts anti-apoptosis effects, maintains cell barrier function, and reduces anti-oxidative stress in hypoxia-induced human brain microvascular endothelial cells (HBMECs) under hypoxic conditions by regulating miR-6867-5p/TWIST1 (Yan et al., 2020).

Opa-interacting protein 5 antisense RNA 1 (OIP5-AS1) is involved in the development of multiple human cancers (Deng et al., 2018; Wang et al., 2019). OIP5-AS1 could also function through miRNAs. Overexpressing OIP5-AS1 attenuates oxidative stress and inflammation in a middle cerebral artery occlusion/reperfusion (MCAO/R) rat model, possibly *via* antioxidant functions activated by targeting miR-186-5p to increase C1q/TNF-related protein 3 (CTRP3) and Nrf2 (Chen et al., 2021b). Similarly, neuroprotective effects were observed from the lncRNA CCAAT enhancer binding protein α antisense RNA 1 (CEBPA-AS1).

OGD/R upregulates CEBPA-AS1 expression in SH-SY5Y cells, while CEBPA-AS1 silencing antagonizes the effects of OGD/R on oxidative stress by decreasing ROS levels and increasing SOD and GSH levels. These results indicate that CEBPA-AS1 knockdown reduces OGD/R-induced oxidative stress in neurons (Di et al., 2021).

The lncRNA potassium voltage-gated channel subfamily Q member 1 opposite strand 1 (KCNQ1OT1) aggravates oxidative stresses and inflammation during hypoxia. KCNQ1OT1 could target miRNAs to alter oxidative stress. For example, KCNQ1OT1 is upregulated in blood samples from patients with AIS. In OGD/R model PC12 cells, the cells were protected from oxidative stress injury. KCNQ1OT1 might target miR-140-3p to enhance hypoxia-inducible factor-1α (HIF-1α) expression (Yi et al., 2020).

The lncRNA ZFAS1 is significantly downregulated in patients with AIS. Mechanistically, lncRNA ZFAS1 acts as a “sponge” for miR-582-3p, which upregulates nitric oxide synthase 3 (NOS3) expression and associated antioxidant functions (Zhang and Zhang, 2020).

LncRNA small nucleolar RNA host gene16 (SNHG16) has been well-documented for oncogenic properties in various malignancies (Gong et al., 2020). X-linked inhibitor-of-apoptosis protein (XIAP) suppresses neurological dysfunction and neuronal apoptosis, thereby relating to preconditioning treatment for cerebral I/R injury (Wang et al., 2021b). Overexpressing SNHG16 enhances cell proliferation and inhibits apoptosis. SNHG16 might enhance XIAP expression by sponging miR-421 to attenuate cell inflammation and oxidative stress in an OGD/R-induced SK-N-SH cell model (Cao et al., 2022).

The lncRNA growth-arrest-specific transcript 5 (GAS5) is widely reported as a tumor suppressor gene (Saussez et al., 1998). Suppressing GAS5 exerts anti-oxidative stress effects in MCAO rats, downregulates GAS5-impaired NOS activity, reduces MDA and protein carbonyl content, and enhances SOD, CAT, and glutathione peroxidase (GSH-Px) activities. Additionally, GAS5 increases cell viability and decreases apoptosis in OGD/R-induced PC12 cells and MCAO rats (Wu et al., 2021).

Previous studies indicate that some antioxidants (CAT and SOD) and pro-oxidants (LDH, MDA, and NO) could be potential endogenous targets for stroke therapy (Chen et al., 2011). In MCAO mice, silencing lncRNA Gm11974 contributes to the recovery of neurological function. In addition, depleting Gm11974 suppresses neuronal apoptosis in OGD-stimulated N2a cells, These neuroprotective effects may occur because Gm11974 silencing increases SOD and CAT activity while decreasing MDA, LDH, and NO levels (Yang et al., 2021).

Cyclic AMP response element binding protein (CREB), a leucine zipper transcription factor, inhibits ROS production and inhibits severe ischemic injury by upregulating brain-derived neurotrophic factor (BDNF) and Bcl-2 (Kitagawa, 2007). The role of lncRNA AK046177 has not been elucidated, though in MCAO rats, inhibiting AK046177 expression significantly alleviates I/R- or OGD/R-mediated neuronal injury. This neuroprotective effect after cerebral ischemia injury occurs *via* AK046177 inhibition, while increases cAMP synthesis, promotes CREB expression and phosphorylation, stimulates Nrf2 activation, and attenuates I/R- or OGD/R- mediated injury (Wang et al., 2020a).

The lncRNA SNHG7 is downregulated in OGD-treated neurons. SNHG7 expression reduces OGD-induced cell damage. Increased SNHG7 expression reverses N2a cell viability during OGD, promotes SOD and CAT activity, and decreases OGD-triggered MDA and ROS production. FGF9, a target of miR-134-5p, represses OGD/R-induced cell damage by promoting cell viability and repressing cell apoptosis (Gao et al., 2020). SNHG7 overexpression protects against OGD-induced neuronal damage by modulating cytotoxicity, cell viability, apoptosis, and oxidative stress. The underlying molecular mechanism is that SNHG7 stimulates FGF9 expression by associating with miR-134-5p (Sun et al., 2021).

In summary, multiple lncRNAs are involved in the pathogenesis of AIS through oxidative stress. Regulating lncRNAs expression has neuroprotective effects in AIS cells and animal models. In addition, most of these lncRNAs regulate the expression of their downstream mRNAs by sponging their target miRNAs, which regulates oxidative stress (Zhang et al., 2020c). These recent studies provide a new mechanism for lncRNAs to participate in AIS pathogenesis and suggest potential novel therapeutic strategies for AIS.

### LncRNAs Mediating Oxidative Stress in Alzheimer’s Disease

Oxidative stress plays an important role in AD pathogenesis. Excessive oxidative stress causes lipid peroxidation, protein nitrification, and nucleic acid destruction, which affects the synaptic capacity of neurons and even leads to apoptosis (Jiang et al., 2016a). When amyloid (Aβ) is irreversibly deposited in brain tissue, oxygen free radicals are produced, which causes oxidative stress, resulting in neuronal dysfunction, metabolic disorder, and a significant decline in learning, cognition, and memory (Cheignon et al., 2018). Aβ activates *N*-methyl-D-aspartic acid receptors to promote oxygen free radical production, thereby inducing neuronal damage (Shelat et al., 2008). *In vitro* cultured neurons exposed to Aβ have increased lipid peroxidation and hydrogen peroxide levels. Antioxidants may inhibit Aβ aggregation (Meske et al., 2008). In addition, Aβ can inactivate antioxidant enzymes, which induces ROS generation and forms a positive feedback pathway to aggravate oxidative stress in the nervous system (Dessalew et al., 2007). Moreover, oxidative stress activates glycogen synthetic kinase (GSK-3), which is an isomer of Tau protein kinase. Therefore, oxidative stress also promotes Tau protein phosphorylation, resulting in neurological impairment (Rönnemaa et al., 2008).

LncRNAs may antagonize Aβ neurotoxicity through various mechanisms, including antioxidant activity. In hippocampal neurons, Aβ25-35 treatment induces oxidative stress, which is indicated by significantly decreased SOD and GSH-Px activity and increased MDA levels. XIST knockdown alleviates the effects of Aβ25-35 treatment on SOD, GSH-Px, and MDA levels. Studies investigating the underlying mechanism by which XIST functions suggest that XIST exerts regulatory functions by binding to miR-132 and upregulating its expression. Importantly, miR-132 has been reported to play a neuroprotective role against oxidative stress (Wong et al., 2013; Wang et al., 2018b).

Aβ25-35 upregulates H19 and downregulates miR-129 in PC12 cells. Further, silencing H19 and upregulating miR-129 improves cell viability and represses apoptosis in PC12 cells stimulated by Aβ25-35 in AD. This protective effect was partly achieved by two ncRNAs, elevated SOD and CAT expression, and decreased MDA expression (Zhang et al., 2021).

The lncRNA BDNF-AS, a natural antisense transcript to BDNF, negatively modulates BDNF *in vitro* and *in vivo* (Modarresi et al., 2012). In Aβ25-35-induced PC12 cells, ROS production is significantly increased, MDA activity is substantially elevated, and SOD and CAT activity are dramatically decreased. Silencing BDNF-AS reverses the Aβ25-35 induced oxidative stress response, suggesting that BDNF-AS may participate in AD development and progression *via* oxidative stress (Guo et al., 2018). A previous study suggests that BDNF protects neurons against 3-nitropropionic acid-induced oxidative stress by increasing sestrin2 expression and decreasing ROS formation (Wu et al., 2016). Therefore, silencing BDNF-AS may exert anti-oxidative effects by increasing BDNF expression.

Wilms’ tumor suppressor (WT1) is highly expressed in AD and promote apoptosis, which leads to neurological failure (Lovell et al., 2003). The lncRNA WT1-AS, a natural antisense transcript to WT1, negatively modulates WT1. In Aβ25-35-induced SH-SY5Y cells, WT1-AS is downregulated. In contrast, overexpressing WT1-AS significantly decreases ROS, MDA, and LDH levels in SH-SY5Y cells, while SOD and GSH-Px are markedly elevated. miR-375, which is a target of WT1, targets the 3′ UTR of SIX4. Overexpressing WT1-AS inhibits miR-375 expression by suppressing WT1, which prevents AD occurrence and development (Wang et al., 2020b).

LncRNA taurine upregulated gene 1 (TUG1) functions by regulating miRNA expression. TUG1 silencing strengthens antioxidant ability and depresses neuronal apoptosis in an Aβ25-35-induced AD mouse model. Further, TUG1 might perform antioxidant functions by targeting miR-15a to inhibit Rho-associated protein kinase 1 (ROCK1) (Li et al., 2020).

Although several studies confirm that oxidative stress is an important mechanism of AD pathogenesis, the number of studies on lncRNAs that regulate oxidative stress during AD pathogenesis is limited. In Aβ25-35-induced AD cells and animal models, XIST, H19, BDNF-As, WT1-As, and TUG1 regulate oxidative stress by modulating target miRNA or *via* other pathways. By investigating the regulatory mechanism of these lncRNAs, we will further understand the role of lncRNAs in AD pathogenesis and identify potential therapeutic strategies for AD.

### LncRNAs Mediating Oxidative Stress in Parkinson’s Disease

Parkinson’s disease is a progressive age-related neurodegenerative disease (Andrei Surguchov, 2022). The main pathological features of PD are the loss of dopaminergic neurons in the substantia nigra dense region and the formation of Lewy bodies (Samii et al., 2004). The main clinical manifestations of PD are static tremor, myotonia, bradykinesia, and abnormal posture and gait. These symptoms are often accompanied by non-motor symptoms such as anxiety, depression, and cognitive decline (Khoo et al., 2013; Lopiano et al., 2016). The pathogenesis of PD is still unclear. Recent studies have confirmed that oxidative stress plays an important role in PD occurrence and development (Gaki and Papavassiliou, 2014). Oxidative stress damages neurons through free radicals. Substantia nigra dopaminergic neurons are particularly susceptible to oxidative stress (Rodriguez-Pallares et al., 2012). ROS target mitochondria, resulting in dysfunction and reduced energy production. Dopaminergic neurons in the substantia nigra have super-long unmyelinated axons and high energy consumption (Pissadaki and Bolam, 2013). In patients with PD, substantia nigra dopamine neurons under oxidative stress have energy demands that exceeds the energy supply, which eventually kills the damaged neurons. In the remaining dopaminergic neurons, cellular metabolism is accelerated due to compensatory dopamine synthesis, which produces more free radicals and further increases oxidative stress (Dias et al., 2013). Furthermore, oxidative deamination of dopamine by monoamine oxidase (MAO) produces H_2_O_2_ as a by-product, while enzymatic oxidation of the electron-rich catechin portion of dopamine by tyrosinase, cycloxase, and other enzymes produces O_2_^–^ (Muñoz et al., 2012). Spontaneous dopamine oxidation can also occur through interactions with unstable iron and other biomaterials, thereby producing ROS (H_2_O_2_, O_2_^–^, and OH) (Sun et al., 2018). Considering the significant role of oxidative stress in PD pathogenesis, ameliorating oxidative stress by regulating lncRNAs can deepen our understanding of PD pathogenesis and elucidate new treatments.

LncRNA myocardial infarction-associated transcript (MIAT) was originally isolated as a candidate gene for myocardial infarction. The over-expanding role of MIAT in various human diseases has been recently revealed, including PD (Shen et al., 2021). MIAT is highly expressed in 1-methyl-4-phenyl-1,2,3,6-tetrahydropyridine (MPTP)-treated mice and 1-methyl-4-phenylpyridinium ion (MPP +)-treated cells. Downregulating MIAT promotes SOD and GSH production, but inhibits MDA in MPP + -treated cells. Blocking MIAT increases cell viability and inhibits cell apoptosis. Additionally, MIAT enhances Nrf2 expression by modulating its target, miR-221-3p (Lang et al., 2022).

The lncRNA non-coding RNA activated by DNA damage (NORAD) is a highly conserved lncRNA that is necessary for genome stability. Dysregulated NORAD is present in various cancer types (Soghli et al., 2021). In MPP + -induced neuroblastoma cells, reduced NORAD expression is observed. Interestingly, NORAD overexpression relieves cytotoxicity and inflammatory responses of neuroblastoma cells after MPP + treatment. NORAD upregulation also inhibits MPP + -induced LDH increase, reduces increased ROS activity, and suppresses SOD activity in SK-N-SH and SK-N-AS cells. The inhibition of oxidative stress by NORAD upregulation is partly achieved by regulating the miR-204-5p/solute carrier family 5-member 3 (SLC5A3) axis (Zhou et al., 2020).

In MPTP-induced PD rat models, silencing RMST also has anti-oxidative stress effects. Silencing RMST could reduce inflammatory responses and suppress neuron apoptosis in the substantia nigra of PD rats. In addition, silencing RMST increases SOD, CAT, and GSH-Px activity, reduces NOS activity, and decreases MDA and NO content. These results suggest that RMST could be neuroprotective against dopamine neurons injury caused by oxidative stress (Ma et al., 2021).

miR-205-5p plays a protective role during PD. Upregulation miR-205-5p inhibits the expression of leucine-rich repeat kinase 2 (LRRK2) and prevents neurite outgrowth defects induced by the R1441G LRRK2 mutation, which indicates a major role of miR-205-5p in neuron survival (Cho et al., 2013). AL049437 is a “sponge” or an endogenous competitor of miR-205-5p. Silencing AL049437 promotes the protective function of miR-205-5p, suppresses apoptosis of SH-SY5Y cells, and reduces oxidative stress (Zhang et al., 2020b).

As an activator of the antioxidant pathway, overexpression of MALAT1 was observed in MPTP-treated PD mice. MALAT1 contributes to inflammasome activation in microglial cells, which triggers neuronal injury by interacting with enhancer of zeste homolog 2 (EZH2) to regulate Nrf2-mediated antioxidative responses. Reducing MALAT1 increases antioxidant capacity and attenuates oxidative stress damage (Cai et al., 2020).

Long non-coding RNA-p21 (lnc-p21) regulates mRNA translation, gene expression, protein stability, and p53-dependent apoptosis (Hall et al., 2015). Lnc-p21 is upregulated in PD and remains overexpressed during PD progression (Kraus et al., 2017). In human neuroblastoma SH-SY5Y cells treated with MPP +, lnc-p21 is highly expressed. Knocking down lnc-p21 mitigates MPP +-induced oxidative stress and neuroinflammation, as evidenced by decreased ROS generation, increased SOD activity, and decreased interleukin 6 (IL-6), tumor necrosis factor α (TNF-α) and IL-1β levels. TRPM2 is activated by oxidative stress, resulting in elevated intracellular Ca^2+^ concentrations (Nazıroğlu, 2011). Mechanistically, lnc-p21 knockdown exerts anti-oxidative function by downregulating TRPM2 expression *via* sponging miR-625, the target of lnc-p21 (Ding et al., 2019).

Gene microarray analysis revealed decreased lncRNA-T199678 expression in an exogenous α-synuclein-induced SH-SY5Y cellular model of PD (Lin et al., 2018). In α-synuclein-induced SH-SY5Y cellular PD models, ROS levels are significantly increased, while overexpressing lncRNA-T199678 reverses intracellular oxidative stress induced by exogenous α-synuclein. miR-101-3p is a potential target for lncRNA-T199678 and binds with α-synuclein at a specific 3′ UTR binding site. Therefore, overexpressing lncRNA-T199678 inhibits α-synuclein-induced neuronal damage *via* regulating intracellular oxidative stress, cell cycle dysfunction, and apoptosis by targeting miR-101-3p (Bu et al., 2020).

*In vitro* and *in vivo* studies of PD suggest that lncRNAs regulate miRNA participation in oxidative stress through endogenous competition mechanisms and directly regulate SOD and LDH levels. Moreover, Nrf2 is a main target of lncRNA regulation. Therefore, lncRNAs may be involved in regulating oxidative stress in PD through various mechanisms and may become a potential target for PD treatment.

### LncRNAs Mediating Oxidative Stress in Spinal Cord Injury

Spinal cord injury is a common CNS disorder that is characterized by different degrees of sensorimotor dysfunction, which often leads to paraplegia, quadriplegia, and other pathological changes that seriously affect the quality of life in a patient (James et al., 2019). Oxidative stress plays an important role in SCI pathogenesis. Previous studies showed that abundant ROS are generated immediately after SCI, which can induce oxidative stress not neutralized in time. More importantly, oxidative stress is involved in secondary events, such as excitatory toxicity, inflammatory responses, and neuronal and glial apoptosis (Fatima et al., 2015). Taking effective measures to prevent or decrease oxidative stress after injury is an effective measure to intervene in SCI. For each link of free radical chain reactions, an appropriate inhibitor or free radical scavenger can achieve effective anti-oxidation (Kikuchi et al., 2013). At present, various antioxidants have been studied. Some results suggest that lncRNAs may affect oxidative stress through various mechanisms and play a therapeutic role in SCI.

LncRNA cancer susceptibility candidate 9 (CASC9) has been extensively studied in various cancers and functions as an oncogene in bladder cancer and esophageal squamous cell carcinoma (Huo et al., 2020; Zhao et al., 2020). Lipopolysaccharide (LPS) downregulates CASC9 and upregulates its derived miR-383-5p in LPS-induced PC12 cells. CASC9-derived miR-383-5p targets the 3′ UTR of LDHA to downregulate Nrf2 and HO-1 proteins. CASC9 also exert functions through miRNAs. Overexpressing CASC9 attenuates oxidative stress and inflammation in an SCI rat model. This antioxidant function may occur by targeting miR-383-5p to inhibit Nrf2/HO-1 signaling (Guan and Wang, 2021).

lncRNA growth arrest specific transcript 5 (GAS5) regulates the development of some CNS disorders. For instance, GAS5 was identified as a potential tumor suppressor in glioma (Zhao et al., 2015). Overexpressing GAS5 facilitates cell apoptosis induced by OGD in MCAO mouse models by reducing mitogen activated protein kinase 4 (MAP4K4) expression (Deng et al., 2020). The CELF2/VAV1 pathway might coordinate with GAS5 to enhance OGD-triggered oxidative stress and cell injury. GAS5 knockdown downregulates VAV1 expression by recruiting CELF2 to the coding region of VAV1 mRNA, which mitigates SCI by reducing oxidative stress and caspase-3 activity in rat models (Wang et al., 2021a).

LncRNA tectonic family member 2 (TCTN2) is a functional RNA that is involved in autophagy in neurons, thereby modulating neuronal apoptosis and improving SCI (Ren et al., 2019). Insulin-like growth factor 1 receptor (IGF1R) has an active role in neural stem cell-mediated motor recovery after spinal cord transection (Hwang et al., 2018). In the spinal cord of SCI model rats and LPS-stimulated PC12 cells, increased miR-329-3p levels are observed. Inhibiting miR-329-3p reverses LPS-induced neuronal apoptosis, oxidative stress, and inflammation by upregulating IGF1R. miR-329-3p is a target of TCTN2. Thus, exosomes derived from TCTN2-modified mesenchymal stem cells may improve SCI *via* the miR-329-3p/IGF1R axis (Liu et al., 2022).

LncRNA SOX2 overlapping transcript (SOX2OT) is linked to the development of multiple human cancers (Zhang et al., 2016; Zhan et al., 2020). Neurod1 is involved in SCI-stimulated oxidative stress and inflammatory damage (Fu et al., 2018). SOX2OT was identified as a miR-331-3p sponge that positively regulates Neurod1 expression. SOX2OT knockdown ameliorates LPS-mediated inflammation, improves cell viability, reduces apoptosis, and inhibits oxidative stress in PC12 cells by modulating the miR-331-3p/Neurod1 axis and activating Janus kinase signaling (Li et al., 2021b).

### LncRNAs Mediating Oxidative Stress in Other Disorders

Oxidative stress is closely related to cancer cell survival and the development of glioma (Gilbert et al., 2014). Excessive ROS levels produced by oxidative stress can affect cell function and change gene expression, which may promote glioma progression (Kim et al., 2014). LncRNA H19 encodes a 2.3-kb long transcript and is a carcinogenic lncRNA in several cancers, including glioma (Jiang et al., 2016b). Overexpressing H19 in gliomas can contribute to malignant transformation, promote tumor proliferation, invasion, infiltration, and chemoresistance (Jia et al., 2016). In glioma cell models, H19 levels are induced by oxidative stress and are increased in U251 and LN229 cells. Meanwhile, overexpressing H19 in U251 and LN229 cells causes temozolomide resistance, which indicates that H19 confers temozolomide resistance to glioma cells (Duan et al., 2018).

Epilepsy is a disease in which epigenetic mechanisms play an important role in the pathogenesis (Surguchov et al., 2017). Temporal lobe epilepsy (TLE) is a common CNS disorder that is characterized by recurrent seizures (Chen et al., 2016). LncRNA maternally expressed gene 3 (MEG3) is a well-known tumor suppressor gene (Wang et al., 2012). In TLE rat models, MEG3 expression is downregulated along with high MDA content and decreased SOD activity. Upregulating MEG3 enhances cell viability, reverses oxidative stress, and inhibits apoptosis through activating the PI3K/AKT/mTOR pathway in TLE. These findings may contribute to developing new therapeutic targets for epilepsy (Zhang et al., 2020a).

## Conclusion

The cellular and molecular changes underlying CNS injuries provide a wealth of potential biomarkers and therapeutic targets. Oxidative stress plays an important role in the occurrence and development of CNS disorders. In addition, secondary pathological damage caused by oxidative stress, including neuroinflammatory responses, mitochondrial damage, and increased apoptosis, are the main cause of exacerbated CNS disorders. Therefore, regulating oxidative stress in CNS disorders may help alleviate neurological damage and provide a new therapeutic strategy for CNS disorders.

**FIGURE 5:**
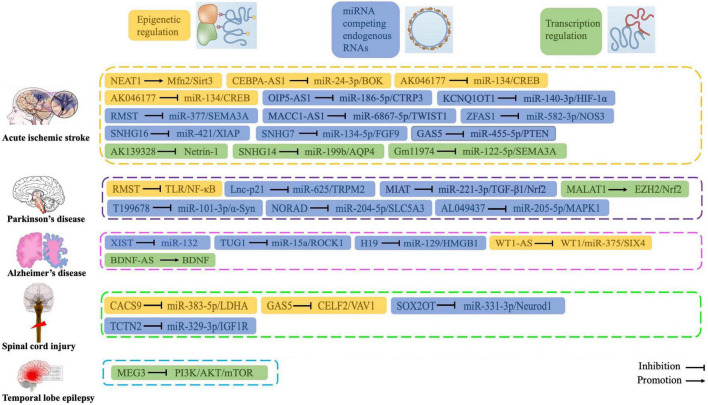
Roles and functions of lncRNAs in regulating oxidative stress during central nervous system disorders.

In recent years, accumulating studies show that lncRNAs, which were originally considered “junk” and “noise,” are widely involved in multiple human diseases, including CNS disorders and are associated with oxidative stress. We demonstrate that many lncRNAs regulate oxidative stress by interacting with miRNAs, thereby regulating SOD activity, MDA expression levels, and other functions. In addition, some lncRNAs directly regulate antioxidant pathways (such as Nrf2/HO-1 signaling) to regulate the pathogenesis of CNS disorders. These oxidative stress-related lncRNAs may be potential key biomarkers and therapeutic targets of CNS disorders (Figure 5). Further, these lncRNAs may provide a novel strategy for disease diagnosis and treatment. Future studies will elucidate the precise mechanisms by which lncRNAs regulate oxidative stress in CNS disorders.

## Author Contributions

YZ and XX wrote the manuscript. YZ produced the figures. XX edited and revised the review. Both authors have read and approved the final manuscript.

## Conflict of Interest

The authors declare that the research was conducted in the absence of any commercial or financial relationships that could be construed as a potential conflict of interest.

## Publisher’s Note

All claims expressed in this article are solely those of the authors and do not necessarily represent those of their affiliated organizations, or those of the publisher, the editors and the reviewers. Any product that may be evaluated in this article, or claim that may be made by its manufacturer, is not guaranteed or endorsed by the publisher.
